# Causal Link between Inflammatory Bowel Disease and Fistula: Evidence from Mendelian Randomization Study

**DOI:** 10.3390/jcm12072482

**Published:** 2023-03-24

**Authors:** Zongbiao Tan, Shijie Zhu, Chuan Liu, Yang Meng, Jiao Li, Jixiang Zhang, Weiguo Dong

**Affiliations:** 1 Department of Gastroenterology, Renmin Hospital of Wuhan University, 238 Jiefang Road, Wuhan 430060, China; 2 Department of Occupational and Environmental Health, School of Public Health, Wuhan University, Wuhan 430071, China; 3 Department of Ophthalmology, Renmin Hospital of Wuhan University, 238 Jiefang Road, Wuhan 430060, China

**Keywords:** inflammatory bowel disease, fistula, genetic epidemiology, Mendelian randomization

## Abstract

Background: Previous observational studies have found that fistulas are common in Crohn’s disease (CD) and less common in ulcerative colitis (UC). However, some patients have a fistula before diagnosis. Based on retrospective analysis, it was not possible to determine whether there was a bi-directional causal relationship between inflammatory bowel disease (IBD) and fistulas. Methods: Data were extracted from the open GWAS database; 25,042 cases and 34,915 controls were included for IBD, and 6926 cases and 30,228 controls were included for fistula. Two-sample Mendelian randomization and multivariable Mendelian randomization were used in combination to determine the causal relationship between IBD and fistula. Results: Forward MR showed that IBD increased the risk of colonic or urogenital fistula (FISTULA) (OR: 1.09, 95% CI: 1.05 to 1.13, *p* = 1.22 × 10^−6^), mainly associated with fissure and fistula of the anal and rectal regions (FISSANAL) (OR:1.10, 95% CI:1.06 to 1.14, *p* = 6.12 × 10^−8^), but not with fistulas involving the female genital tract (FEMGENFISTUL) (OR:0.97, 95% CI: 0.85 to 1.11, *p* = 0.669). Furthermore, both UC and CD increased the risk of FISTULA. However, after adjusting by MVMR, only CD increased the risk of FISTULA (OR: 1.06, 95% CI: 1.02 to 1.11, *p* = 0.004), and UC did not increase the risk of FISTULA (OR: 1.01, 95% CI: 0.95 to 1.06, *p* = 0.838). Reverse MR showed that fistulas did not increase the risk of IBD. Conclusion: Our study confirms it is CD, rather than UC, that casually leads to an increased risk of fistula, but fistulas do not increase the risk of IBD.

## 1. Introduction

Inflammatory bowel disease (IBD), including ulcerative colitis (UC) and Crohn’s disease (CD), is a chronic idiopathic disease characterized by intestinal inflammation. In recent years, IBD has become a global disease with higher incidence and prevalence in the course of industrialization [1,2]. IBD is associated with a variety of complications [3], such as strictures [4], fistulas [5], infections [6], and cancers [7], which bring a heavy economic burden to society. Due to long-term chronic inflammation of the intestine, abnormal connections, known as fistulas, may form between the intestine and the skin or adjacent organs, including the bladder and vagina. Based on the location of fistulas and the relationship between fistulas and neighboring organs, fistulas can be divided into two primary types: internal fistulas that open to neighboring organs, such as enteric-intestinal fistulas, enteric-bladder fistulas, and ileocolonic fistulas; and external fistulas that open on the body surface, such as enteric-skin fistulas and perianal fistulas [8,9]. Fistula is one of the major intestinal complications of Crohn’s disease (CD), occurring in 35% to 53% of CD patients during their natural course [10]. Perianal fistula is the main type, accounting for about 20–65%, and some patients are treated with perianal fistula as the first symptom [11]. In contrast, the incidence of anal fistula in the European general population was only 18.37 (95% CI: 18.20–18.55%) per 10,000 [12]. In addition, approximately 5 to 10% of women with Crohn’s disease have been reported to develop fistulas involving the genital tract, called genital fistulas [13]. There has been no large-scale epidemiological investigation into the risk of fistula in UC. However, results from a Korean follow-up study of 944 UC patients showed that the cumulative incidence of UC patients with a perianal disease (PAD) in 5 and 10 years was 8.1% and 16.0%, respectively, and the cumulative incidence of perianal sepsis (abscess or fistula) in 5 and 10 years was 2.2% and 4.5%, respectively [14]. Another study also showed that the incidence of perianal fistula in UC patients after ileoanal pouch anastomosis was 9%, which was greatly increased compared with the general population [15]. Fistula can cause recurrent infection and fecal incontinence, which seriously reduces the quality of life of patients. Although previous observational studies have observed an association between IBD and fistulas, it is not possible to ascertain whether there is a causal relationship between the two because of the limitations of observational studies. Different genotypes determine different intermediate phenotypes. If the phenotype represents an individual’s exposure characteristics, the association effect between genotypes and diseases can be used to simulate the effect of exposure factors on diseases. Based on this idea, Mendelian randomization (MR) finds suitable genetic variants to estimate the causal relationship between exposure and outcome. MR looks for valid genotype instrumental variables (IVs) from the genome-wide association studies (GWAS). These IVs need to satisfy three assumptions: (I) strong association with exposure factors; (II) no association with confounders; and (III) association with outcomes only through exposure, which were used to reveal causal relationships between inferred exposure and outcomes [16,17]. Compared with randomized controlled trials (RCTs), MR is less affected by various confounders and is more economical and efficient. Therefore, MR has been widely used in the study of causality inference in recent years. An MR Study confirmed the causal relationship between depression and IBD, indicating that the occurrence of IBD involves the participation of mental and psychological factors [18]. In addition, the causal association between IBD and a variety of diseases, such as psoriasis [19], atopic dermatitis [20], rheumatoid arthritis [21], and so on, was clarified through MR.

To date, there have been no MR Studies on IBDs and fistulas to reveal a causal relationship. In this study, the causal relationship between IBD (UC or CD) and fistula was explored by two-sample MR, and the confounding bias caused by the presence of a common gene locus between UC and CD was removed by multivariable Mendelian randomization (MVMR). This study provides new insight into a causal link between IBD (UC or CD) and fistulas.

## 2. Materials and Methods

**Figure 1:**
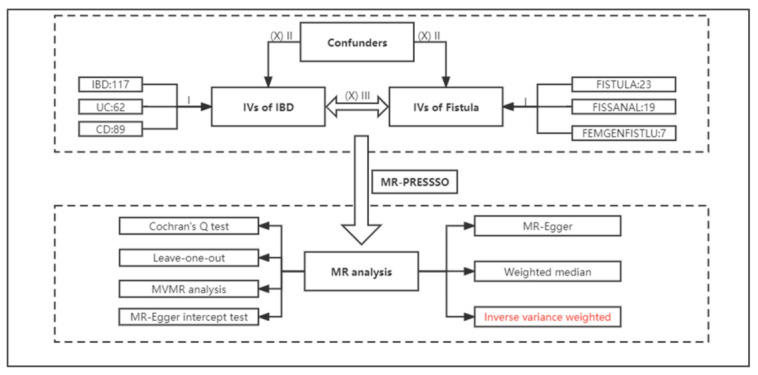
The workflow of this study.

Figure 1 illustrates the conceptual and analytical flow of this study.

### 2.1. Data Sources

In this study, we retrieved IBD data from the IEU Open GWAS database, including IBD, UC, and CD. The IBD data included 25,042 patients and 34,915 controls, and the UC and CD data included 12,366 and 12,194 patients, respectively [22]. These diagnoses are based on accepted radiological, endoscopic, and histopathological criteria [23]. Although European and non-European cohorts were included, the direction and magnitude of the effects of most IBD risk loci were consistent across different populations. Fistula data were obtained from the recently published FinnGen7 database (http://www.finngen.fi) [24]. Data were accessed on 3 November 2022.Population inclusion was selected primarily based on the 10th Edition of the International Classification of Diseases (ICD-10) diagnosis at the time of discharge or cause of death. Colonic or urogenital fistula (FISTULA) included 6926 patients, including the diagnosis of ICD-10 K31.6, K38.3, and K60 (Supplementary Table S1 lists the specific diagnosis names). Fissure and fistula of anal and rectal regions (FISSANAL) included 6610 patients with a primary diagnosis of ICD-10 K60, and fistula involving the female genital tract (FEMGENFISTUL) data included 327 patients with a primary diagnosis of ICD-10 N82. Table 1 shows details of the information for the cohort population.

### 2.2. Selection of Instrumental Variables

When selecting IVs for IBD (UC or CD), firstly, to satisfy the correlation between SNP and exposure, SNP was considered to be strongly correlated with IBD (UC, CD) only when *p*-value <5 × 10^−8^. At the same time, we set the standard as r^2^ = 0.001 and the width of the linkage disequilibrium (LD) area = 10,000 kb to eliminate LD. Secondly, SNPs associated with outcomes (*p* < 5 × 10^−8^) were excluded when combining information from the outcome datasets [25]. Finally, to ensure that the SNPs mentioned above did not produce weak instrumental bias, the formula (F = β^2^/SE^2^) was used to calculate the F value of each SNP, and SNPs with F value < 10 were eliminated. β referred to the effect value of exposure and SE referred to the standard error for effect values of exposure. Only SNPs that met the above criteria were included as IVs in the analysis. In addition, we uploaded the above SNPs to the Phenoscanner Database (www.phenoscanner.medschl.cam.ac.Uk, the search was conducted on 7 November 2022) to check the presence of the second phenotype, which may lead to potential confounding. However, because UC and CD share some common gene loci, we cannot remove all SNPs closely related to both. Therefore, we conducted MVMR to remove the mutual bias between UC and CD. According to the above criteria, there are fewer IVs related to fistula, so we relaxed the criteria for SNP selection, i.e., *p*-value < 5 × 10^−6^, and other conditions remained unchanged.

### 2.3. MR Analyses

Weighted median (WM), MR-Egger, and inverse variance weighted (IVW) methods were used in the bi-directional MR analysis to determine the causal relationship between IBD (UC or CD) and fistula (FISTULA, FISSANAL, and FEMGENFISTUL). WM requires at least 50% of SNPs to meet the conditions of the three assumptions, while MR-Egger relaxes the pleiotropy hypothesis but is prone to weak instrumental bias [26,27]. IVW was used as the primary outcome as it allowed all SNPs to display a random degree of horizontal pleiotropy, providing a more accurate estimate than the two methods described above. To confirm the reliability of our results, we performed separate tests for heterogeneity and pleiotropy. First of all, Cochran’s Q test was conducted to determine whether the selected IVs were heterogeneous. When heterogeneity existed, MR-PRESSO was used for analysis, and the analysis was performed again after removing the outlier IVs. Second, in the process of MR analysis, we used the MR-Egger intercept test to evaluate the possible bias caused by gene pleiotropy due to the use of multiple IVs for causal inference. Finally, the effect of each IV on the results was detected by leave-one-out analysis. MVMR can be used to analyze the causality of multiple exposure factors imposed by a genetic tool on the same outcome variable. The bias caused by confounding can be reduced by MVMR [28]. Therefore, we performed MVMR analysis between UC and CD as two exposures and fistula to remove confounding bias between the two.

### 2.4. Statistics Analysis and Visualization

This study was a secondary analysis of published data, and no changes were made to the original data. All statistical analyses and visualizations were completed in R (version 4.1.3) by 23 November 2022. ‘’TwoSampleMR” and “MRPRESSO” packages were used for MR analyses, and the “forest plot” package was used for visualization. In the process of analysis, IBD (UC or CD) and fistula (FISTULA, FISSANAL, and FEMGENFISTUL) were analyzed several times, so the MR analysis results to determine the causal effect of IBD on fistula were only considered statistically significant when Bonferroni corrected *p*-value < 0.0028 (0.05/18).

## 3. Results

### 3.1. Selection of Instrumental Variables

There were 117 SNPs (IBD), 89 SNPs (CD), and 62 SNPs (UC) with *p* < 5 × 10^−8^. Then, removing SNPs for being palindromic with intermediate allele frequencies, 9576 and 51 IVs representing IBD, CD, and UC were obtained for MR analysis (Supplementary Table S2). In addition, we performed MRPRESSO testing in each direction and found the following outlier IVs conditions: IBD to FISTULA, rs186239; IBD to FISSANAL, rs186239; UC to FISTULA, rs9271176; and UC to FISSANAL, rs9271176. These outlier IVs were removed and reanalyzed. For fistula-related IVs, 20 (FISTULA), 15 (FISSANAL), and 6 (FEMGENFISTUL) IVs were received for follow-up analysis, and the MRPRESSO test did not detect outlier IVs (Supplementary Table S3). All of the IV F values > 10, which ensured that there would be no bias caused by weak IVs in MR analysis.

### 3.2. Forward Mendelian Randomization Analyze

**Figure 2:**
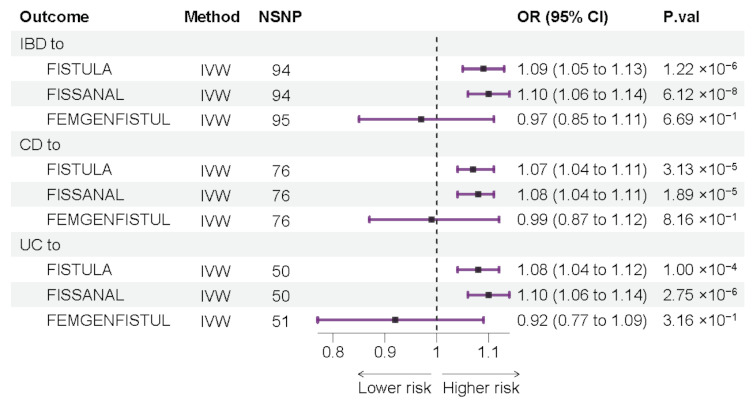
Mendelian randomization (MR) results of IVW. Causal estimation of IBD effects on fistulas (IBD: inflammatory bowel disease; UC: ulcerative colitis; CD: Crohn’s disease; FISTULA: colonic or urogenital fistula; FISSANAL: fissure and fistula of anal and rectal region; FEMGENFISTUL: fistula involving female genital tract; NSNP: the number of single nucleotide polymorphisms used in MR analysis; OR: odds ratio; CI: confidence interval).

Generally, the results of the three methods to determine a causal association between IBD (UC or CD) and fistula (FISTULA, FISSANAL, and FEMGENFISTUL) were not consistent. The results of IVW showed that the causal relationship between IBD and FISTULA was significant (OR: 1.09, 95% CI: 1.05 to 1.13, *p* = 1.22 × 10^−6^), mainly increasing the risk of FISSANAL (OR: 1.10, 95% CI: 1.06 to 1.14, *p* = 6.12 × 10^−8^) and not related to the occurrence of FEMGENFISTUL (OR: 0.97, 95% 0.85 to 1.11, *p* = 0.67 (Figure 2). Further analysis of two subtypes showed that UC and CD increased the risk of FISTULA (UC: OR: 1.08, 95% CI: 1.04 to 1.12, *p* = 1.89 × 10^−5^; CD: OR: 1.07, 95% CI: 1.04 to 1.11, *p* = 3.13 × 10^−5^). Similarly, UC and CD only showed a positive causal relationship for FISSANAL (UC: OR: 1.10, 95% CI: 1.06 to 1.14, *p* = 2.75 × 10^−6^; CD: OR: 1.08, 95% CI: 1.04 to 1.11, *p* = 1.89 × 10^−5^), which was not correlated with FEMGENFISTUL (UC: OR: 0.92, 95% CI: 0.77 to 1.09, *p* = 0.32; CD: OR: 0.99, 95% CI: 0.87 to 1.12, *p* = 0.82) (Figure 2). The above results were equally significant after correcting the *p*-value using the Bonferroni method. The results of WM also showed significant causal associations between IBD (including UC and CD) and the fistula (including FISTULA and FISSANAL), with all *p* values < 0.05 (Supplementary Table S4). Only the relationship between IBD and fistula (FISTULA and FISSANAL) was significant among the causal associations estimated by the MR-Egger method. The estimated effect size of the genetically predicted IBD (UC or CD) on increased fistula (FISTULA, FISSANAL, and FEMGENFISTUL) is shown in the scatterplot (Supplementary Figure S1). Although heterogeneity was observed in some of the results after the Cochran Q test, heterogeneity was acceptable with random effects IVW analysis as the main result. The *p*-values for the MR-Egger intercept were all >0.05, indicating that there was no interference caused by pleiotropy (Supplementary Table S4). In addition, “leave-one-out” analysis did not detect the existence of outlier IVs, which also confirmed the robustness of our results (Supplementary Figure S2).

### 3.3. Forward Multivariable Mendelian Randomization Analyze

**Figure 3:**
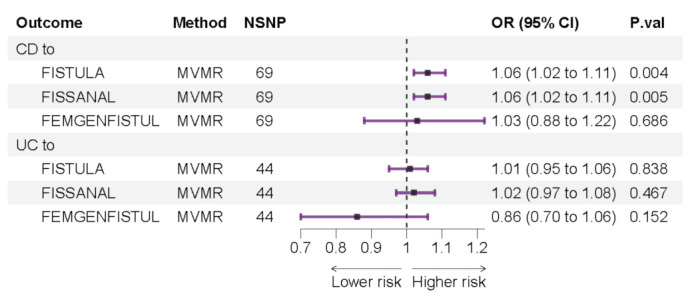
Multivariable Mendelian randomization (MVMR) results. Causal estimation of IBD subtype effects on fistulas (IBD: inflammatory bowel disease; UC: ulcerative colitis, CD: Crohn’s disease; FISTULA: colonic or urogenital fistula; FISSANAL: fissure and fistula of anal and rectal regions; FEMGENFISTUL: fistula involving female genital tract; NSNP: the number of single nucleotide polymorphisms used in MR analysis; OR: odds ratio; CI: confidence interval).

Based on the above results, both UC and CD seem to be related to the occurrence of fistulas. However, considering the possible confounding effect of CD in UC, we further conducted MVMR analysis of the causal effect of UC and CD on fistula. The results showed that only CD was significantly associated with fistula (OR: 1.06, 95% CI: 1.02 to 1.11, *p* = 0.004), while UC did not increase the risk of fistula (OR: 1.01, 95% CI: 0.95 to 1.06, *p* = 0.84) (Figure 3). After fistula classification, CD increased the risk of FISSANAL (OR: 1.06, 95% CI: 1.02 to 1.11, *p* = 0.005) and was not associated with FEMGENFISTUL (OR: 1.03, 95% CI: 0.88 to 1.22, *p* = 0.69) (Figure 3). The occurrence of FISSANAL and FEMGENFISTUL was not affected by UC (FISSANAL: OR: 1.02, 95% CI: 0.97 to 1.08, *p* = 0.47; FEMGENFISTUL: OR: 0.86, 95% CI: 0.70 to 1.06, *p* = 0.15) (Figure 3).

### 3.4. Reverse Mendelian Randomization Analyze

**Figure 4:**
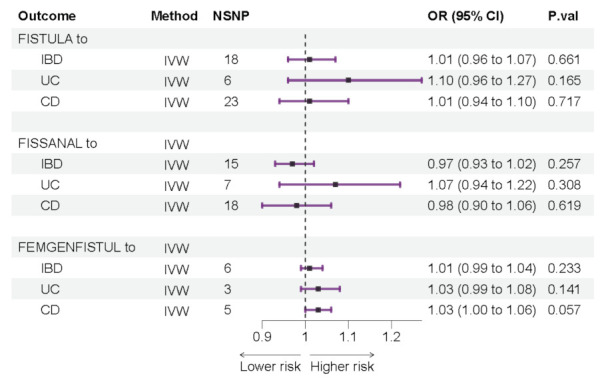
Mendelian randomization (MR) results of IVW. Causal estimation of fistula effects on IBD (IBD: inflammatory bowel disease; UC: ulcerative colitis; CD: Crohn’s disease; FISTULA: colonic or urogenital fistula; FISSANAL: fissure and fistula of anal and rectal regions; FEMGENFISTUL: fistula involving female genital tract; NSNP: the number of single nucleotide polymorphisms used in MR analysis; OR: odds ratio; CI: confidence interval).

We explored the impact of fistula on IBD as an exposure factor. Results showed that FISTULA would not increase the risk of IBD (UC and CD) occurrence: FISTULA to IBD (OR: 1.01, 95% CI: 0.96 to 1.07, *p* = 0.66; FISTULA to UC (OR: 1.10, 95% CI: 0.96 to 1.27, *p* = 0.17); FISTULA to CD (OR: 1.01, 95% CI: 0.94 to 1.1, *p* = 0.72) (Figure 4). Similarly, FISSANAL had no significant effect on IBD (UC and CD) (FISSANAL to IBD: OR: 0.97, 95% CI: 0.93 to 1.02, *p* = 0.26, FISSANAL to UC: OR: 1.07, 95% CI: 0.94 to 1.23, *p* = 0.31, FISSANAL to CD: OR: 0.98, 95% CI: 0.90 to 1.06, *p* = 0.62) (Figure 4). In addition, FEMGENFISTUL was not associated with IBD (FEMGENFISTUL to IBD: OR: 1.01, 95% CI: 0.99 to 1.04, *p* = 0.23, FEMGENFISTUL to UC: OR: 1.03, 95% CI: 0.99 to 1.08, *p* = 0.14, FEMGENFISTUL to CD: OR: 1.03, 95% CI: 1.0 0 to 1.06, *p* = 0.06) (Figure 4). Neither the Cochran Q test nor the MR-Egger intercept test could detect the existence of heterogeneity and pleiotropy (Supplementary Table S5). None of the other methods showed significant results, suggesting that fistula (FISTULA, FISSANAL, and FEMGENFISTUL) did not affect the occurrence of IBD (UC, CD). The results of “leave-one-out” analysis and scatterplots are attached (Supplementary Figures S3 and S4).

## 4. Discussion

To our knowledge, this is the first MR study to analyze the causality between IBD and fistula. In this study, we explored the causal relationship between IBD (UC or CD) and fistula (FISTULA, FISSANAL, and FEMGENFISTUL) bi-directionally. Our results, in support of the previous observational study, indicate that fistula is primarily associated with CD. In addition, no adequate evidence that fistula increases the risk of IBD was found in reverse MR analysis.

Fistula is a complication of IBD, which can aggravate the disease and compromise the quality of life of patients. In particular, severe fistulas can cause intestinal damage and eventually require surgical management, increasing the difficulty of IBD management [29,30]. The mechanism of fistula is not well understood but may be related to bacterial infection of the gut and transmural inflammation of the mucosa, leading to infiltration into adjacent organs, tissues, or skin [31,32]. Perianal fistula is the most common of all fistulas, affecting nearly a quarter of the CD population. Our results confirmed a significant positive relationship between CD and fistulas, especially FISSANAL. In addition, although forward MR results showed that UC could also increase the incidence of fistula, it was considered that most IBD-related susceptibility gene loci were simultaneously associated with UC and CD [33]. Therefore, the causal relationship between UC and fistula may be confused by CD. After eliminating the mixing of CD and UC by MVMR, we found that UC did not increase the risk of fistula. The causes of perianal fistula can be summarized as persistent inflammatory irritation, bacterial infection, and epithelialization after wound repair failure [34,35]. First of all, both UC and CD manifest as chronic inflammation involving the intestine. UC is generally limited to the mucosal layer and submucosa of the colon, while CD is more penetrating, often manifesting as fissure-like ulcers, which can reach deep into the submucosa, muscular layer, and even the serous membrane [36]. Furthermore, the damage of the mucosa creates the basic conditions for the formation of fistulas. Regarding the involvement of infectious factors, no clear strain has been found to be associated with fistula development, but the presence of peptidoglycan has been confirmed at the fistula and the use of antibiotics can improve the symptoms of perianal fistula [37,38]. Therefore, experts speculate that fistula may be a severe inflammatory reaction caused by bacterial infection triggering the immune response based on the defect of the intestinal epithelial barrier [39]. To restore normal structure and function, epithelial-mesenchymal transformation (EMT) occurs at the fistula, where myofibroblasts are activated and migrate to the damaged site for repair under the induction of TGF-β [40,41]. At the same time, myofibroblasts secrete matrix metalloproteinase (MMP) to degrade the extracellular matrix, which in turn leads to continuous tissue destruction, resulting in chronic fistula [42]. A rectovaginal fistula is an abnormal connection between the rectum and vagina. The causes of genital fistulas include obstetric trauma, Crohn’s disease, secondary to cryptogenic gland abscess, and radiation injury [43,44]. Ischemic damage to the soft tissues of the pelvis caused by the baby’s head during prolonged labor is the leading cause of genital fistula, especially in countries with poor health care, where up to 5.6 per 1,000 women develop genital fistula after childbirth [45]. Although a survey of patients with Crohn’s disease showed management of rectovaginal fistulas in 9% of all fistulas [43], there was a lack of history regarding whether the patient had a previous obstetric injury. Our results suggest that there is no causal relationship between IBD and FEMGENFISTUL. Therefore, whether Crohn’s disease is a direct factor of genital fistula in patients, or a secondary result of birth canal injury, needs further prospective investigation. Fistula is a risk predictor for CD exacerbations, and accurate assessment of fistula by clinicians is essential for subsequent treatment and management [46]. Fistulas have a significant impact on the quality of life of patients, yet there is currently no effective means of intervention. Therefore, fistulas need to be managed with a proactive attitude, integrated drugs, and a multidisciplinary approach.

The advantage of this study is that there are currently no RCTs of fistula in IBD, and existing clinical observational studies are subject to some unavoidable confounding factors. By using large-scale GWAS data to perform MR analysis on IBD and fistula, we obtained a relatively accurate causal assessment and confirmed the causal relationship between IBD and fistula. In addition, the confounding bias between UC and CD was considered and corrected by MVMR analysis. Of course, certain limitations should be recognized. First of all, it is difficult to predict the risk of different fistulas in CD due to incomplete fistula data and limited fistula classification. Secondly, the incidence of fistula gradually accumulates with the prolongation of the course of the disease, and there are certain differences between sexes. We did not stratify the population. IBD has a course characterized by alternating periods of remission and relapse, and analysis of the impact of IBD disease activity on fistula is lacking. In addition, because the FinnGen database does not provide the corresponding article description, we were unable to adjust for using the same covariates for both samples. These factors may influence the judgment of the results.

## 5. Conclusions

In summary, our results support a potential causal relationship between IBD and fistulas, primarily influenced by CD, and confirm that fistulas are not associated with the occurrence of IBD (UC and CD). Therefore, we should strengthen the management of CD patients to identify fistulas at an early stage and cooperate with multidisciplinary experts to intervene in fistula occurrence.

## Figures and Tables

**Table 1:** Detail of the data for the cohort population. (IBD: inflammatory bowel disease; UC: ulcerative colitis; CD: Crohn’s disease; FISTULA: colonic or urogenital fistula, FISSANAL: fissure and fistula of anal and rectal regions; FEMGENFISTUL, fistula involving female genital tract).

Variable	Number of Cases	Number of Controls	Data Resource	Population	PMID	Year
IBD	25,042	34915	ebi-a-GCST004131	Mix	28067908	2017
UC	12,366	33,609	ebi-a-GCST004132	Mix	28067908	2017
CD	12,194	28,072	ebi-a-GCST004133	Mix	28067908	2017
FISTULA	6926	30,228	finn-b-K11_FISTULA	European	-	2021
FISSANAL	6610	253,186	finn-b-K11_FISSANAL	European	-	2021
FEMGENFISTUL	327	94,394	finn-b-N14_FEMGENFISTUL	European	-	2021

## Data Availability

The datasets for this study can be found in the GWAS database (https://gwas.mrcieu.ac.uk/) and FinnGen database (http://www.finngen.fi). All data were accessed on 3 November 2022.

## Supplementary Materials

The following supporting information can be downloaded at: https://www.mdpi.com/article/10.3390/jcm12072482/s1, Supplementary Table S1. The specific diagnostic name of the fistula. Supplementary Table S2. IBD (UC, CD)-related SNPs. Supplementary Table S3. Fistula-related SNPs. Supplementary Table S4. Forward Mendelian Randomization (MR) results. Supplementary Table S5. Reverse Mendelian Randomization (MR) results. Supplementary Figure S1. Scatterplots of the risk of forward genetic association between inflammatory bowel disease and fistulas by MR. Supplementary Figure S2. The result of forward ‘Leave-one-out’ analysis. Supplementary Figure S3. Scatterplots of the risk of reverse genetic association between inflammatory bowel disease and fistulas by MR. Supplementary Figure S4. The result of reverse ‘Leave-one-out’ analysis.

## References

[1] Ng S.C., Shi H.Y., Hamidi N., Underwood F.E., Tang W., Benchimol E.I., Panaccione R., Ghosh S., Wu J.C.Y., Chan F.K.L. (2017). Worldwide incidence and prevalence of inflammatory bowel disease in the 21st century: A systematic review of population-based studies. Lancet.

[2] Jairath V., Feagan B.G. (2020). Global burden of inflammatory bowel disease. Lancet Gastroenterol. Hepatol..

[3] Kaur M., Dalal R.L., Shaffer S., Schwartz D.A., Rubin D.T. (2020). Inpatient Management of Inflammatory Bowel Disease-Related Complications. Clin. Gastroenterol. Hepatol..

[4] Rieder F., Zimmermann E.M., Remzi F.H., Sandborn W.J. (2013). Crohn’s disease complicated by strictures: A systematic review. Gut.

[5] Geldof J., Iqbal N., LeBlanc J.F., Anandabaskaran S., Sawyer R., Buskens C., Bemelman W., Gecse K., Lundby L., Lightner A.L. (2022). Classifying perianal fistulising Crohn’s disease: An expert consensus to guide decision-making in daily practice and clinical trials. Lancet Gastroenterol. Hepatol..

[6] Kochar B., Cai W., Cagan A., Ananthakrishnan A.N. (2020). Pretreatment Frailty Is Independently Associated With Increased Risk of Infections After Immunosuppression in Patients With Inflammatory Bowel Diseases. Gastroenterology.

[7] Shah S.C., Itzkowitz S.H. (2022). Colorectal Cancer in Inflammatory Bowel Disease: Mechanisms and Management. Gastroenterology.

[8] Thia K.T., Sandborn W.J., Harmsen W.S., Zinsmeister A.R., Loftus E.V. (2010). Risk factors associated with progression to intestinal complications of Crohn’s disease in a population-based cohort. Gastroenterology.

[9] Householder S., Picoraro J.A. (2022). Diagnosis and Classification of Fistula from Inflammatory Bowel Disease and Inflammatory Bowel Disease-Related Surgery. Gastrointest. Endosc. Clin. North Am..

[10] Tjandra D., Garg M., Behrenbruch C., McCormick J., Simkin P., Prentice R., Trinh A., Al-Ani A., Vaughan R., Macrae F. (2021). Review article: Investigation and management of internal fistulae in Crohn’s disease. Aliment. Pharmacol. Ther..

[11] Rackovsky O., Hirten R., Ungaro R., Colombel J.F. (2018). Clinical updates on perianal fistulas in Crohn’s disease. Expert Rev. Gastroenterol. Hepatol..

[12] Sarveazad A., Bahardoust M., Shamseddin J., Yousefifard M. (2022). Prevalence of anal fistulas: A systematic review and meta-analysis. Gastroenterol. Hepatol. Bed Bench.

[13] De la Poza G., Lopez-Sanroman A., Taxonera C., Marín-Jimenez I., Gisbert J.P., Bermejo F., Opio V., Muriel A. (2012). Genital fistulas in female Crohn’s disease patients.: Clinical characteristics and response to therapy. J. Crohn’s Colitis.

[14] Choi Y.S., Kim D.S., Lee D.H., Lee J.B., Lee E.J., Lee S.D., Song K.H., Jung H.J. (2018). Clinical Characteristics and Incidence of Perianal Diseases in Patients With Ulcerative Colitis. Ann. Coloproctology.

[15] Heimann T.M., Swaminathan S., Slater G.I., Kurtz R.J. (2022). Perianal Fistula After Ileoanal Pouch in Patients With Ulcerative Colitis: A Review of 475 Patients Operated on at a Major IBD Center. Dis. Colon Rectum.

[16] Emdin C.A., Khera A.V., Kathiresan S. (2017). Mendelian Randomization. JAMA.

[17] Burgess S., Scott R.A., Timpson N.J., Smith G.D., Thompson S.G., EPIC-InterAct Consortium (2015). Using published data in Mendelian randomization: A blueprint for efficient identification of causal risk factors. Eur. J. Epidemiol..

[18] Luo J., Xu Z., Noordam R., van Heemst D., Li-Gao R. (2022). Depression and Inflammatory Bowel Disease: A Bidirectional Two-sample Mendelian Randomization Study. J. Crohn’s Colitis.

[19] Li Y., Guo J., Cao Z., Wu J. (2022). Causal Association Between Inflammatory Bowel Disease and Psoriasis: A Two-Sample Bidirectional Mendelian Randomization Study. Front. Immunol..

[20] Meisinger C., Freuer D. (2022). Causal Association Between Atopic Dermatitis and Inflammatory Bowel Disease: A 2-Sample Bidirectional Mendelian Randomization Study. Inflamm. Bowel Dis..

[21] Meisinger C., Freuer D. (2022). Rheumatoid arthritis and inflammatory bowel disease: A bidirectional two-sample Mendelian randomization study. Semin. Arthritis Rheum..

[22] De Lange K.M., Moutsianas L., Lee J.C., Lamb C.A., Luo Y., Kennedy N.A., Jostins L., Rice D.L., Gutierrez-Achury J., Ji S.-G. (2017). Genome-wide association study implicates immune activation of multiple integrin genes in inflammatory bowel disease. Nat. Genet..

[23] Lamb C.A., Kennedy N.A., Raine T., Hendy P.A., Smith P.J., Limdi J.K., Hayee B., Lomer M.C.E., Parkes G.C., Selinger C. (2019). British Society of Gastroenterology consensus guidelines on the management of inflammatory bowel disease in adults. Gut.

[24] Kurki M.I., Karjalainen J., Palta P., Sipilä T.P., Kristiansson K., Donner K., Reeve M.P., Laivuori H., Aavikko M., Kaunisto M.A. (2022). FinnGen: Unique genetic insights from combining isolated population and national health register data. medRxiv.

[25] Hartwig F.P., Davies N.M., Hemani G., Davey Smith G. (2016). Two-sample Mendelian randomization: Avoiding the downsides of a powerful, widely applicable but potentially fallible technique. Int. J. Epidemiol..

[26] Bowden J., Smith G.D., Haycock P.C., Burgess S. (2016). Consistent Estimation in Mendelian Randomization with Some Invalid Instruments Using a Weighted Median Estimator. Genet. Epidemiol..

[27] Bowden J., Davey Smith G., Burgess S. (2015). Mendelian randomization with invalid instruments: Effect estimation and bias detection through Egger regression. Int. J. Epidemiol..

[28] Sanderson E. (2021). Multivariable Mendelian Randomization and Mediation. Cold Spring Harb. Perspect. Med..

[29] Agrawal M., Spencer E.A., Colombel J.F., Ungaro R.C. (2021). Approach to the Management of Recently Diagnosed Inflammatory Bowel Disease Patients: A User’s Guide for Adult and Pediatric Gastroenterologists. Gastroenterology.

[30] Scharl M., Rogler G. (2014). Pathophysiology of fistula formation in Crohn’s disease. World J. Gastrointest. Pathophysiol..

[31] Lee M.J., Parker C.E., Taylor S.R., Guizzetti L., Feagan B.G., Lobo A.J., Jairath V. (2018). Efficacy of Medical Therapies for Fistulizing Crohn’s Disease: Systematic Review and Meta-analysis. Clin. Gastroenterol. Hepatol..

[32] Vogel J.D., Johnson E.K., Morris A.M., Paquette I.M., Saclarides T.J., Feingold D.L., Steele S.R. (2016). Clinical Practice Guideline for the Management of Anorectal Abscess, Fistula-in-Ano, and Rectovaginal Fistula. Dis. Colon Rectum.

[33] Guan Q. (2019). A Comprehensive Review and Update on the Pathogenesis of Inflammatory Bowel Disease. J. Immunol. Res..

[34] Gecse K., Khanna R., Stoker J., Jenkins J.T., Gabe S., Hahnloser D., D’Haens G. (2013). Fistulizing Crohn’s disease: Diagnosis and management. United Eur. Gastroenterol. J..

[35] Tozer P.J., Lung P., Lobo A.J., Sebastian S., Brown S.R., Hart A.L., Fearnhead N., On behalf of ENiGMA Collaboration (2018). Review article: Pathogenesis of Crohn’s perianal fistula-understanding factors impacting on success and failure of treatment strategies. Aliment. Pharmacol. Ther..

[36] Schofield J.B., Haboubi N. (2020). Histopathological Mimics of Inflammatory Bowel Disease. Inflamm. Bowel Dis..

[37] Van Onkelen R., Mitalas L., Gosselink M., van Belkum A., Laman J., Schouten W. (2013). Assessment of microbiota and peptidoglycan in perianal fistulas. Diagn. Microbiol. Infect. Dis..

[38] Thia K.T., Mahadevan U., Feagan B.G., Wong C., Cockeram A., Bitton A., Bernstein C.N., Sandborn W.J. (2009). Ciprofloxacin or metronidazole for the treatment of perianal fistulas in patients with Crohn’s disease: A randomized, double-blind, placebo-controlled pilot study. Inflamm. Bowel Dis..

[39] Siegmund B., Feakins R.M., Barmias G., Ludvig J.C., Teixeira F.V., Rogler G., Scharl M. (2016). Results of the Fifth Scientific Workshop of the ECCO (II): Pathophysiology of Perianal Fistulizing Disease. J. Crohn’s Colitis.

[40] Boros E., Nagy I. (2019). The Role of MicroRNAs upon Epithelial-to-Mesenchymal Transition in Inflammatory Bowel Disease. Cells.

[41] Lovisa S., Genovese G., Danese S. (2019). Role of Epithelial-to-Mesenchymal Transition in Inflammatory Bowel Disease. J. Crohn’s Colitis.

[42] Leeb S.N., Vogl D., Gunckel M., Kiessling S., Falk W., Göke M., Schölmerich J., Gelbmann C.M., Rogler G. (2003). Reduced migration of fibroblasts in inflammatory bowel disease: Role of inflammatory mediators and focal adhesion kinase. Gastroenterology.

[43] Das B., Snyder M. (2016). Rectovaginal Fistulae. Clin. Colon Rectal Surg..

[44] Ngongo C.J., Raassen T., Mahendeka M., Lombard L., van Roosmalen J., Temmerman M. (2022). Rare causes of genital fistula in nine African countries: A retrospective review. BMC Womens Health.

[45] Ngongo C.J., Raassen T., Mahendeka M., Lombard L., van Roosmalen J., Temmerman M. (2022). A retrospective review of genital fistula occurrence in nine African countries. BMC Pregnancy Childbirth.

[46] Kotze P.G., Shen B., Lightner A., Yamamoto T., Spinelli A., Ghosh S., Panaccione R. (2018). Modern management of perianal fistulas in Crohn’s disease: Future directions. Gut.

