# Supplementary material for: Causal Link between Inflammatory Bowel Disease and Fistula: Evidence from Mendelian Randomization Study
Source: J Clin Med. 2023 Mar 24;12(7):2482. doi: 10.3390/jcm12072482 (PMC10095427; doi:10.3390/jcm12072482)
Supplement: Supplementary file 1 [file jcm-12-02482-s001.zip › Supplementary materials.pdf]

Supplementary materials

Supplementary Table S1. The specific diagnostic name of the fistula(FISTULA: Colonic or urogenital fistula, FISSANAL: Fissure and fistula of anal and rectal regions, FEMGENFISTUL Fistula involving female genital tract)

Supplementary Table S2. IBD (UC, CD) related SNPs (IBD: Inflammatory bowel disease, UC: Ulcerative colitis, CD: Crohn's disease,)

Supplementary Table S3. Fistula related SNPs (FISTULA: Colonic or urogenital fistula, FISSANAL: Fissure and fistula of anal and rectal regions, FEMGENFISTUL Fistula involving female genital tract)

Supplementary Table S4. Forward Mendelian Randomization (MR) results. (IBD: Inflammatory bowel disease, UC: Ulcerative colitis, CD: Crohn's disease, FISTULA: Colonic or urogenital fistula, FISSANAL: Fissure and fistula of anal and rectal regions, FEMGENFISTUL Fistula involving female genital tract, NSNP: the number of single nucleotide polymorphisms used in MR analysis, OR: odds ratio, CI: confidence interval)

Supplementary Table S5. Reverse Mendelian Randomization (MR) results. (IBD: Inflammatory bowel disease, UC: Ulcerative colitis, CD: Crohn's disease, FISTULA: Colonic or urogenital fistula, FISSANAL: Fissure and fistula of anal and rectal regions, FEMGENFISTUL Fistula involving female genital tract, NSNP: the number of single nucleotide polymorphisms used in MR analysis, OR: odds ratio, CI: confidence interval)

Supplementary Figure S1. Scatter plots of the risk of forward genetic association between inflammatory bowel disease and fistulas by MR.(The slopes of each line represent the causal association for each method. IBD: Inflammatory bowel disease, UC: Ulcerative colitis, CD: Crohn's disease, FISTULA: Colonic or urogenital fistula, FISSANAL: Fissure and fistula of anal and rectal regions, FEMGENFISTUL Fistula involving female genital tract)

Supplementary Figure S2. The result of forward 'Leave-one-out'.

Supplementary Figure S3. Scatter plots of the risk of reverse genetic association between inflammatory bowel disease and fistulas by MR.(The slopes of each line represent the causal association for each method. IBD: Inflammatory bowel disease, UC: Ulcerative colitis, CD: Crohn's disease, FISTULA: Colonic or urogenital fistula, FISSANAL: Fissure and fistula of anal and rectal regions, FEMGENFISTUL Fistula involving female genital tract)

Supplementary Figure S4. The result of reverse 'Leave-one-out'.
