# Supplementary material for: Causal Link between Inflammatory Bowel Disease and Fistula: Evidence from Mendelian Randomization Study
Source: J Clin Med. 2023 Mar 24;12(7):2482. doi: 10.3390/jcm12072482 (PMC10095427; doi:10.3390/jcm12072482)
Supplement: Supplementary file 1 [file jcm-12-02482-s001.zip › Supplementary table S1.pdf]

Data	ID	Classification of diseases
FISTULA	ICD-10 K31.6	Gastric and duodenal fistula
	ICD-10 K38.3	Appendiceal fistula
	ICD-10 K60	Fissure and fistula of anal and rectal regions
	ICD-10 N32.1	Bladder fistula
	ICD-10 N82.2	A fistula from the vagina to the small intestine
	ICD-10 N82.3	A fistula from the vagina to the large intestine
	ICD-10 N82.4	Other female entero-genital fistulas
	ICD-9 537.4	Fistula of stomach or duodenum
	ICD-9 565	Anal fissure and fistula
	ICD-8 565	Anal fissure and fistula
	ICD-8 54302	Other diseases of appendix
FISSANAL	ICD-10 K60	Fissure and fistula of anal and rectal regions
FEMGENFISTUL	ICD-10 N82	Fistula involving female genital tract
