# Supplementary material for: Causal Link between Inflammatory Bowel Disease and Fistula: Evidence from Mendelian Randomization Study
Source: J Clin Med. 2023 Mar 24;12(7):2482. doi: 10.3390/jcm12072482 (PMC10095427; doi:10.3390/jcm12072482)
Supplement: Supplementary file 1 [file jcm-12-02482-s001.zip › Supplementary table S2.pdf]

Supplementary table S2. (1) SNPs related to IBD; (2) SNPs related to UC; (3) SNPs related to CD.

(1)

ID	SNP	Effect_allele	Other_allele	Se	Beta	Chr	P_val	F
ebi-a-GCST004131	rs12136659	C	T	0.014	0.087	1	1.02E-09	37.537
ebi-a-GCST004131	rs2488398	C	G	0.015	0.099	1	3.63E-11	43.702
ebi-a-GCST004131	rs10746475	A	T	0.016	0.131	1	1.58E-15	63.610
ebi-a-GCST004131	rs4654925	C	G	0.012	-0.117	1	4.80E-21	89.333
ebi-a-GCST004131	rs112936798	C	A	0.033	-0.184	1	2.89E-08	30.849
ebi-a-GCST004131	rs35730213	C	G	0.014	-0.135	1	7.50E-22	92.434
ebi-a-GCST004131	rs3024493	A	C	0.017	0.191	1	4.04E-31	134.139
ebi-a-GCST004131	rs11209013	G	A	0.012	0.077	1	4.46E-10	38.861
ebi-a-GCST004131	rs11581607	A	G	0.029	-0.658	1	4.59E-111	500.603
ebi-a-GCST004131	rs1336900	A	G	0.013	-0.085	1	2.98E-11	43.891
ebi-a-GCST004131	rs10800309	G	A	0.013	-0.123	1	1.94E-20	85.528
ebi-a-GCST004131	rs1268339	C	T	0.016	0.091	1	2.75E-08	30.963
ebi-a-GCST004131	rs1317209	A	G	0.016	0.116	1	3.79E-13	52.926
ebi-a-GCST004131	rs3820330	A	C	0.014	-0.089	1	1.72E-10	40.595
ebi-a-GCST004131	rs4276914	A	G	0.013	0.078	1	3.15E-10	39.238
ebi-a-GCST004131	rs7532133	G	A	0.013	0.079	1	3.83E-09	34.669
ebi-a-GCST004131	rs11677002	C	T	0.013	-0.093	2	1.37E-13	54.596
ebi-a-GCST004131	rs55946629	A	C	0.018	0.130	2	5.45E-13	52.000
ebi-a-GCST004131	rs4676408	A	G	0.013	0.101	2	7.63E-15	60.481
ebi-a-GCST004131	rs7608697	C	A	0.013	0.140	2	1.67E-28	122.577
ebi-a-GCST004131	rs13422838	C	T	0.021	-0.114	2	2.56E-08	31.087

ebi-a-GCST004131	rs62180107	C	G	0.013	-0.080	2	1.55E-09	36.456
ebi-a-GCST004131	rs3792111	T	C	0.012	0.139	2	5.12E-29	125.838
ebi-a-GCST004131	rs1558619	T	G	0.012	-0.084	2	8.90E-12	46.973
ebi-a-GCST004131	rs76286777	C	T	0.015	0.100	2	4.65E-11	43.508
ebi-a-GCST004131	rs72852162	C	A	0.020	-0.113	2	2.30E-08	31.238
ebi-a-GCST004131	rs6740847	G	A	0.013	-0.092	2	1.22E-13	54.642
ebi-a-GCST004131	rs62183956	T	C	0.013	-0.078	2	4.49E-10	38.938
ebi-a-GCST004131	rs1131095	C	T	0.013	0.164	3	1.22E-35	155.773
ebi-a-GCST004131	rs56116661	T	C	0.016	-0.100	3	9.27E-10	37.638
ebi-a-GCST004131	rs77272631	C	G	0.042	0.229	3	3.72E-08	30.237
ebi-a-GCST004131	rs503734	G	A	0.012	-0.069	3	2.67E-08	31.144
ebi-a-GCST004131	rs2593855	T	C	0.014	-0.083	3	2.54E-09	35.318
ebi-a-GCST004131	rs11734570	A	G	0.013	0.069	4	4.80E-08	29.861
ebi-a-GCST004131	rs62324212	A	C	0.013	0.089	4	2.67E-12	48.670
ebi-a-GCST004131	rs4957256	T	C	0.016	-0.118	5	3.37E-14	57.858
ebi-a-GCST004131	rs17656349	T	C	0.013	0.073	5	5.17E-09	34.199
ebi-a-GCST004131	rs6579807	T	C	0.019	0.125	5	4.01E-11	43.742
ebi-a-GCST004131	rs1445004	T	C	0.013	0.169	5	3.48E-40	176.869
ebi-a-GCST004131	rs62378712	C	T	0.014	-0.078	5	4.23E-08	29.864
ebi-a-GCST004131	rs6873866	C	T	0.013	-0.092	5	6.15E-13	51.548
ebi-a-GCST004131	rs10041497	C	T	0.013	0.082	5	1.95E-10	40.308
ebi-a-GCST004131	rs755374	T	C	0.013	0.177	5	1.59E-39	173.886
ebi-a-GCST004131	rs56235845	G	T	0.014	0.088	5	1.77E-10	40.387
ebi-a-GCST004131	rs11739135	C	G	0.013	0.137	5	1.10E-27	119.421
ebi-a-GCST004131	rs341295	T	C	0.012	0.070	5	1.45E-08	32.050
ebi-a-GCST004131	rs11152949	G	A	0.013	0.102	6	1.56E-14	58.701

ebi-a-GCST004131	rs1267496	C	G	0.016	0.105	6	3.39E-11	43.859
ebi-a-GCST004131	rs145568234	G	T	0.048	0.860	6	4.73E-73	326.425
ebi-a-GCST004131	rs6457681	T	G	0.015	-0.169	6	3.75E-28	121.576
ebi-a-GCST004131	rs4712528	C	G	0.015	0.104	6	7.14E-12	47.085
ebi-a-GCST004131	rs143210366	G	T	0.036	0.284	6	3.14E-15	62.059
ebi-a-GCST004131	rs62408218	T	C	0.013	-0.082	6	2.40E-10	40.209
ebi-a-GCST004131	rs212402	A	G	0.013	-0.074	6	1.06E-08	32.666
ebi-a-GCST004131	rs34140409	T	C	0.024	-0.158	6	2.28E-11	44.613
ebi-a-GCST004131	rs6933404	C	T	0.015	0.086	6	6.64E-09	33.547
ebi-a-GCST004131	rs35171809	G	A	0.012	0.109	6	1.16E-18	78.243
ebi-a-GCST004131	rs10953551	G	A	0.013	-0.103	7	4.94E-16	66.160
ebi-a-GCST004131	rs243505	G	A	0.013	-0.081	7	3.04E-10	39.552
ebi-a-GCST004131	rs149169037	A	G	0.024	-0.134	7	3.26E-08	30.569
ebi-a-GCST004131	rs1456896	T	C	0.013	0.088	7	4.50E-11	43.679
ebi-a-GCST004131	rs62482552	A	G	0.013	-0.074	7	1.97E-08	31.651
ebi-a-GCST004131	rs11768365	G	A	0.015	-0.084	7	3.88E-08	30.322
ebi-a-GCST004131	rs78771661	T	C	0.067	-0.385	8	8.95E-09	33.084
ebi-a-GCST004131	rs4380956	A	G	0.013	0.091	8	1.12E-12	51.004
ebi-a-GCST004131	rs938650	A	G	0.019	-0.107	8	1.41E-08	32.291
ebi-a-GCST004131	rs1887428	C	G	0.013	-0.164	9	2.46E-36	157.301
ebi-a-GCST004131	rs10114470	C	T	0.014	0.148	9	4.10E-27	115.916
ebi-a-GCST004131	rs3829110	G	A	0.013	0.157	9	3.52E-36	158.558
ebi-a-GCST004131	rs1250573	A	G	0.014	-0.098	10	1.11E-12	50.431
ebi-a-GCST004131	rs10826797	T	G	0.014	-0.099	10	3.99E-13	52.990
ebi-a-GCST004131	rs6584282	G	A	0.012	-0.152	10	1.19E-34	150.260
ebi-a-GCST004131	rs11195128	T	C	0.013	0.079	10	2.74E-09	35.461

ebi-a-GCST004131	rs2384352	G	A	0.013	0.095	10	3.12E-13	52.701
ebi-a-GCST004131	rs10761659	G	A	0.013	0.159	10	2.30E-36	158.240
ebi-a-GCST004131	rs7918084	T	C	0.013	0.071	10	1.38E-08	32.262
ebi-a-GCST004131	rs111456533	A	G	0.017	-0.103	10	1.18E-09	36.781
ebi-a-GCST004131	rs11221335	C	T	0.015	0.083	11	2.44E-08	31.224
ebi-a-GCST004131	rs11236797	A	C	0.013	0.149	11	7.19E-33	141.705
ebi-a-GCST004131	rs11066188	A	G	0.013	0.087	12	1.76E-11	45.200
ebi-a-GCST004131	rs117981694	A	G	0.041	0.345	12	4.53E-17	70.544
ebi-a-GCST004131	rs12825700	A	G	0.013	0.132	12	1.27E-25	108.685
ebi-a-GCST004131	rs3897234	C	T	0.015	0.097	13	1.90E-11	44.844
ebi-a-GCST004131	rs140933577	C	T	0.031	-0.186	13	1.13E-09	37.070
ebi-a-GCST004131	rs194746	T	C	0.012	0.083	14	1.84E-11	45.128
ebi-a-GCST004131	rs3850378	C	T	0.021	0.154	14	1.10E-13	55.061
ebi-a-GCST004131	rs1864239	G	A	0.178	1.337	15	6.27E-14	56.258
ebi-a-GCST004131	rs56062135	T	C	0.015	0.138	15	1.37E-21	90.841
ebi-a-GCST004131	rs7190426	C	A	0.016	-0.087	16	2.06E-08	31.650
ebi-a-GCST004131	rs28374519	A	G	0.014	-0.111	16	6.55E-16	65.055
ebi-a-GCST004131	rs9934775	T	C	0.017	-0.112	16	8.77E-11	42.099
ebi-a-GCST004131	rs8056255	A	T	0.033	0.277	16	2.99E-17	71.498
ebi-a-GCST004131	rs11548656	G	A	0.036	-0.237	16	5.18E-11	43.008
ebi-a-GCST004131	rs749910	A	G	0.014	0.196	16	7.83E-46	201.928
ebi-a-GCST004131	rs2301127	A	G	0.013	0.078	16	4.96E-10	38.617
ebi-a-GCST004131	rs16940202	C	T	0.017	0.113	16	2.50E-11	44.708
ebi-a-GCST004131	rs12936409	T	C	0.012	0.141	17	7.73E-30	128.566
ebi-a-GCST004131	rs744166	G	A	0.013	-0.111	17	1.34E-18	77.468
ebi-a-GCST004131	rs714910	C	A	0.014	-0.096	17	6.23E-12	47.600

ebi-a-GCST004131	rs113846785	CG	C	0.018	-0.132	17	2.47E-13	53.696
ebi-a-GCST004131	rs1319951	G	C	0.015	-0.085	18	7.50E-09	33.514
ebi-a-GCST004131	rs80262450	A	G	0.019	0.158	18	1.04E-16	69.240
ebi-a-GCST004131	rs4807569	C	A	0.015	0.128	19	4.24E-17	71.025
ebi-a-GCST004131	rs7256518	A	G	0.028	-0.167	19	1.63E-09	36.392
ebi-a-GCST004131	rs62126610	G	A	0.017	0.141	19	2.60E-17	71.841
ebi-a-GCST004131	rs11669299	T	C	0.016	-0.111	19	1.84E-12	49.716
ebi-a-GCST004131	rs6062496	A	G	0.013	0.137	20	2.83E-26	112.788
ebi-a-GCST004131	rs4256018	G	T	0.014	0.079	20	1.23E-08	32.440
ebi-a-GCST004131	rs6017342	C	A	0.014	0.116	20	1.07E-17	73.324
ebi-a-GCST004131	rs6063502	G	A	0.013	-0.073	20	4.55E-08	30.004
ebi-a-GCST004131	rs154873	A	G	0.013	-0.081	20	7.38E-10	37.934
ebi-a-GCST004131	rs1297264	G	A	0.013	-0.146	21	3.98E-31	134.634
ebi-a-GCST004131	rs2836881	T	G	0.015	-0.164	21	1.96E-29	126.640
ebi-a-GCST004131	rs2838517	C	T	0.013	-0.128	21	1.83E-24	104.858
ebi-a-GCST004131	rs2413583	T	C	0.017	-0.173	22	4.60E-24	102.590
ebi-a-GCST004131	rs5754100	C	T	0.016	0.129	22	7.14E-16	65.307
ebi-a-GCST004131	rs5763793	T	G	0.013	0.073	22	1.47E-08	31.879

(2)

ID	SNP	Effect_allele	Other_allele	Se	Beta	Chr	P_val	F
ebi-a-GCST004133	rs7544646	G	C	0.016	-0.117	1	2.53E-13	53.290
ebi-a-GCST004133	rs3024493	A	C	0.021	0.210	1	7.46E-24	100.959
ebi-a-GCST004133	rs3820330	A	C	0.018	-0.159	1	3.91E-19	79.490
ebi-a-GCST004133	rs11209026	A	G	0.036	-0.483	1	1.99E-41	182.024
ebi-a-GCST004133	rs6658353	C	G	0.016	-0.157	1	1.17E-22	96.163

ebi-a-GCST004133	rs7554511	A	C	0.018	-0.145	1	4.27E-16	66.175
ebi-a-GCST004133	rs7523335	A	G	0.021	-0.139	1	3.42E-11	43.749
ebi-a-GCST004133	rs2816954	A	T	0.023	0.138	1	1.80E-09	36.052
ebi-a-GCST004133	rs1317209	A	G	0.020	0.182	1	2.90E-19	80.204
ebi-a-GCST004133	rs79051659	A	G	0.026	0.161	1	1.30E-09	36.961
ebi-a-GCST004133	rs4654925	C	G	0.016	-0.222	1	2.61E-44	194.418
ebi-a-GCST004133	rs7608697	C	A	0.016	0.160	2	3.03E-23	98.392
ebi-a-GCST004133	rs55905347	A	G	0.017	0.105	2	2.09E-10	40.315
ebi-a-GCST004133	rs62180181	T	C	0.017	0.123	2	8.08E-13	51.403
ebi-a-GCST004133	rs4676408	A	G	0.017	0.143	2	1.19E-17	73.631
ebi-a-GCST004133	rs1811711	G	C	0.022	-0.130	2	6.09E-09	33.932
ebi-a-GCST004133	rs1131095	C	T	0.017	0.159	3	2.18E-21	89.911
ebi-a-GCST004133	rs755374	T	C	0.017	0.171	5	9.73E-24	100.468
ebi-a-GCST004133	rs72704802	T	C	0.021	-0.122	5	2.89E-09	35.247
ebi-a-GCST004133	rs17715902	A	G	0.017	0.097	5	4.62E-09	34.427
ebi-a-GCST004133	rs6889364	A	G	0.023	0.132	5	7.87E-09	33.417
ebi-a-GCST004133	rs17656349	T	C	0.016	0.090	5	1.54E-08	32.040
ebi-a-GCST004133	rs67111717	G	A	0.017	0.094	5	3.27E-08	30.476
ebi-a-GCST004133	rs9260809	G	A	0.018	-0.110	6	1.96E-09	35.870
ebi-a-GCST004133	rs9267798	C	G	0.028	0.249	6	6.54E-19	78.829
ebi-a-GCST004133	rs9271176	G	A	0.017	-0.350	6	4.20E-91	408.133
ebi-a-GCST004133	rs28383224	G	A	0.017	-0.147	6	4.65E-19	79.156
ebi-a-GCST004133	rs3734851	A	G	0.058	0.503	6	6.58E-18	74.273
ebi-a-GCST004133	rs13200059	A	G	0.044	0.294	6	1.48E-11	45.593
ebi-a-GCST004133	rs6933404	C	T	0.019	0.149	6	2.69E-15	62.477
ebi-a-GCST004133	rs113986290	T	C	0.053	-0.307	6	7.59E-09	33.339

ebi-a-GCST004133	rs798506	C	T	0.018	-0.121	7	1.47E-11	45.393
ebi-a-GCST004133	rs4728142	A	G	0.016	0.100	7	3.23E-10	39.658
ebi-a-GCST004133	rs989960	T	C	0.016	-0.121	7	3.28E-14	57.570
ebi-a-GCST004133	rs10272963	T	C	0.016	-0.151	7	4.11E-21	89.303
ebi-a-GCST004133	rs1887428	C	G	0.017	-0.167	9	9.65E-24	101.208
ebi-a-GCST004133	rs10817678	A	G	0.017	0.133	9	4.42E-15	61.392
ebi-a-GCST004133	rs3812565	C	T	0.016	0.134	9	6.50E-17	69.618
ebi-a-GCST004133	rs10761659	G	A	0.016	0.128	10	1.33E-15	63.601
ebi-a-GCST004133	rs7911117	G	T	0.024	-0.134	10	1.84E-08	31.529
ebi-a-GCST004133	rs7911680	C	A	0.016	-0.153	10	6.71E-22	91.991
ebi-a-GCST004133	rs2212434	T	C	0.016	0.125	11	2.80E-15	62.003
ebi-a-GCST004133	rs2045241	A	G	0.017	-0.106	11	2.83E-10	39.563
ebi-a-GCST004133	rs12825700	A	G	0.016	0.189	12	7.33E-32	137.661
ebi-a-GCST004133	rs1359946	A	G	0.020	0.157	13	6.58E-15	60.485
ebi-a-GCST004133	rs56062135	T	C	0.018	0.108	15	4.66E-09	34.324
ebi-a-GCST004133	rs11645239	G	C	0.020	-0.117	16	4.14E-09	34.457
ebi-a-GCST004133	rs7203363	A	T	0.019	0.107	16	1.41E-08	32.111
ebi-a-GCST004133	rs16940186	C	T	0.021	0.136	16	2.18E-10	40.210
ebi-a-GCST004133	rs113846785	CG	C	0.023	-0.163	17	1.15E-12	50.478
ebi-a-GCST004133	rs12936409	T	C	0.016	0.137	17	5.62E-18	74.636
ebi-a-GCST004133	rs11651246	G	T	0.022	0.147	17	2.01E-11	45.055
ebi-a-GCST004133	rs10408351	A	G	0.020	0.155	19	2.92E-14	57.581
ebi-a-GCST004133	rs78064630	A	G	0.031	0.176	19	1.08E-08	32.616
ebi-a-GCST004133	rs6062496	A	G	0.016	0.136	20	8.97E-17	69.513
ebi-a-GCST004133	rs6017342	C	A	0.017	0.194	20	3.95E-30	130.766
ebi-a-GCST004133	rs2836881	T	G	0.019	-0.222	21	1.11E-32	142.071

ebi-a-GCST004133	rs2838517	C	T	0.016	-0.118	21	1.78E-13	54.114
ebi-a-GCST004133	rs1736161	A	G	0.016	-0.123	21	2.22E-14	58.081
ebi-a-GCST004133	rs9611131	C	T	0.023	-0.149	22	5.11E-11	43.316
ebi-a-GCST004133	rs4993442	T	G	0.018	-0.099	22	3.54E-08	30.465
ebi-a-GCST004133	rs137845	G	A	0.016	0.101	22	1.50E-10	40.944

(3)

ID	SNP	Effect_allele	Other_allele	Se	Beta	Chr	P_val	F
ebi-a-GCST004132	rs12131079	T	C	0.017	-0.109	1	3.99E-10	39.098
ebi-a-GCST004132	rs35730213	C	G	0.018	-0.117	1	1.17E-10	41.499
ebi-a-GCST004132	rs3122605	A	G	0.023	-0.175	1	1.24E-14	59.297
ebi-a-GCST004132	rs114802258	T	C	0.038	-0.225	1	5.11E-09	34.180
ebi-a-GCST004132	rs4316387	C	T	0.019	-0.129	1	7.74E-12	46.731
ebi-a-GCST004132	rs6679677	A	C	0.029	-0.228	1	1.77E-15	63.275
ebi-a-GCST004132	rs6704109	T	C	0.018	0.175	1	5.10E-22	93.267
ebi-a-GCST004132	rs7517847	G	T	0.017	-0.345	1	5.84E-97	436.430
ebi-a-GCST004132	rs11378157	AG	A	0.019	-0.138	2	9.29E-14	55.321
ebi-a-GCST004132	rs11683692	C	T	0.038	-0.214	2	1.75E-08	31.833
ebi-a-GCST004132	rs4343432	G	A	0.016	0.112	2	3.50E-12	48.054
ebi-a-GCST004132	rs11677002	C	T	0.016	-0.112	2	4.57E-12	47.551
ebi-a-GCST004132	rs34004493	G	A	0.018	0.126	2	2.00E-12	49.392
ebi-a-GCST004132	rs3816234	A	G	0.016	0.270	2	1.51E-62	278.601
ebi-a-GCST004132	rs55946629	A	C	0.023	0.176	2	2.85E-14	57.721
ebi-a-GCST004132	rs7608697	C	A	0.016	0.123	2	4.03E-14	56.850
ebi-a-GCST004132	rs6740847	G	A	0.016	-0.104	2	9.72E-11	41.727
ebi-a-GCST004132	rs1583792	T	C	0.016	-0.088	2	3.26E-08	30.388

ebi-a-GCST004132	rs56116661	T	C	0.021	-0.131	3	5.67E-10	38.300
ebi-a-GCST004132	rs6808936	G	A	0.016	0.090	3	1.93E-08	31.527
ebi-a-GCST004132	rs9836291	A	G	0.017	0.172	3	3.77E-24	102.605
ebi-a-GCST004132	rs2581828	G	C	0.016	-0.094	3	6.46E-09	33.740
ebi-a-GCST004132	rs73243877	G	A	0.021	0.116	4	4.12E-08	30.146
ebi-a-GCST004132	rs13107325	T	C	0.028	0.201	4	1.66E-12	49.891
ebi-a-GCST004132	rs62324212	A	C	0.016	0.106	4	8.02E-11	42.290
ebi-a-GCST004132	rs6579807	T	C	0.024	0.199	5	3.44E-16	66.717
ebi-a-GCST004132	rs755374	T	C	0.017	0.197	5	1.38E-29	128.054
ebi-a-GCST004132	rs6451494	C	T	0.017	0.261	5	8.26E-56	246.263
ebi-a-GCST004132	rs112856973	C	T	0.024	-0.161	5	3.61E-11	44.007
ebi-a-GCST004132	rs6873866	C	T	0.016	-0.131	5	1.35E-15	64.195
ebi-a-GCST004132	rs2188962	T	C	0.016	0.200	5	5.59E-36	156.876
ebi-a-GCST004132	rs181826	A	C	0.017	0.116	5	3.24E-12	48.415
ebi-a-GCST004132	rs1012636	T	G	0.020	0.129	6	7.01E-11	42.513
ebi-a-GCST004132	rs1321859	T	C	0.017	-0.105	6	1.18E-09	37.196
ebi-a-GCST004132	rs73516754	C	A	0.017	0.142	6	4.04E-17	70.898
ebi-a-GCST004132	rs35171809	G	A	0.016	0.157	6	9.07E-23	97.004
ebi-a-GCST004132	rs111281598	C	T	0.032	0.275	6	4.17E-18	75.459
ebi-a-GCST004132	rs6941902	C	T	0.027	0.163	6	2.39E-09	35.649
ebi-a-GCST004132	rs7753014	G	C	0.016	-0.099	6	1.39E-09	36.814
ebi-a-GCST004132	rs145568234	G	T	0.063	0.860	6	4.31E-42	184.668
ebi-a-GCST004132	rs9482770	C	T	0.016	0.099	6	1.01E-09	37.120
ebi-a-GCST004132	rs9501641	T	C	0.043	0.303	6	2.57E-12	49.097
ebi-a-GCST004132	rs9258357	C	T	0.022	-0.118	6	5.00E-08	29.793
ebi-a-GCST004132	rs212409	A	G	0.016	-0.110	6	1.49E-11	45.771

ebi-a-GCST004132	rs9656588	C	T	0.017	0.118	7	8.73E-12	46.760
ebi-a-GCST004132	rs938650	A	G	0.025	-0.175	8	1.65E-12	50.026
ebi-a-GCST004132	rs4380956	A	G	0.017	0.132	8	1.15E-15	64.000
ebi-a-GCST004132	rs79832570	C	T	0.034	0.223	8	8.90E-11	42.174
ebi-a-GCST004132	rs10114470	C	T	0.018	0.169	9	1.76E-21	90.841
ebi-a-GCST004132	rs1887428	C	G	0.017	-0.166	9	8.54E-23	96.481
ebi-a-GCST004132	rs4077515	T	C	0.016	0.185	9	3.14E-30	130.129
ebi-a-GCST004132	rs10884966	A	G	0.017	0.113	10	4.13E-11	43.745
ebi-a-GCST004132	rs61839660	T	C	0.026	0.147	10	1.98E-08	31.635
ebi-a-GCST004132	rs2002695	G	A	0.019	-0.129	10	8.31E-12	46.803
ebi-a-GCST004132	rs10822050	C	T	0.016	0.183	10	2.35E-29	127.188
ebi-a-GCST004132	rs2675670	C	G	0.016	0.107	10	2.89E-11	44.500
ebi-a-GCST004132	rs1148246	T	C	0.017	-0.132	10	2.09E-15	62.761
ebi-a-GCST004132	rs1250573	A	G	0.018	-0.152	10	1.92E-17	72.297
ebi-a-GCST004132	rs6584282	G	A	0.016	-0.166	10	3.44E-25	107.381
ebi-a-GCST004132	rs11236797	A	C	0.016	0.176	11	8.51E-28	119.502
ebi-a-GCST004132	rs28999107	T	G	0.018	0.108	12	1.06E-09	37.018
ebi-a-GCST004132	rs77566919	A	G	0.019	-0.109	12	4.13E-09	34.651
ebi-a-GCST004132	rs34635748	T	C	0.050	0.479	12	1.95E-21	90.476
ebi-a-GCST004132	rs1373904	G	A	0.019	0.141	13	9.11E-14	55.656
ebi-a-GCST004132	rs194746	T	C	0.016	0.098	14	1.24E-09	36.674
ebi-a-GCST004132	rs3850378	C	T	0.027	0.199	14	8.31E-14	55.550
ebi-a-GCST004132	rs72743461	A	C	0.019	0.168	15	2.26E-19	81.096
ebi-a-GCST004132	rs2021511	T	C	0.018	-0.108	16	2.63E-09	35.344
ebi-a-GCST004132	rs42861	G	A	0.017	0.124	16	8.87E-14	55.400
ebi-a-GCST004132	rs2076756	G	A	0.017	0.385	16	1.80E-108	489.579

ebi-a-GCST004132	rs7195228	G	C	0.021	-0.133	16	2.09E-10	40.313
ebi-a-GCST004132	rs72798422	C	T	0.038	0.550	16	6.05E-47	206.923
ebi-a-GCST004132	rs10492862	A	C	0.018	0.107	16	1.26E-09	36.754
ebi-a-GCST004132	rs2948542	G	A	0.016	0.102	17	5.15E-10	38.852
ebi-a-GCST004132	rs714910	C	A	0.018	-0.153	17	2.49E-17	71.547
ebi-a-GCST004132	rs12936409	T	C	0.016	0.143	17	4.31E-19	79.433
ebi-a-GCST004132	rs744166	G	A	0.016	-0.114	17	1.80E-12	49.694
ebi-a-GCST004132	rs80262450	A	G	0.024	0.227	18	1.34E-20	86.399
ebi-a-GCST004132	rs144309607	T	C	0.047	-0.371	19	2.69E-15	62.376
ebi-a-GCST004132	rs62126620	A	G	0.020	0.144	19	8.61E-13	51.325
ebi-a-GCST004132	rs4807570	A	G	0.019	0.181	19	6.03E-21	88.049
ebi-a-GCST004132	rs492602	G	A	0.016	0.108	19	2.33E-11	44.774
ebi-a-GCST004132	rs6062496	A	G	0.017	0.122	20	2.62E-13	53.632
ebi-a-GCST004132	rs3761158	A	G	0.017	-0.110	20	2.65E-11	44.283
ebi-a-GCST004132	rs1297264	G	A	0.016	-0.177	21	1.59E-27	117.782
ebi-a-GCST004132	rs2284553	G	A	0.017	0.128	21	1.14E-14	59.898
ebi-a-GCST004132	rs2838517	C	T	0.016	-0.146	21	2.03E-19	80.778
ebi-a-GCST004132	rs2143178	C	T	0.022	-0.209	22	6.84E-21	87.586
ebi-a-GCST004132	rs5754100	C	T	0.021	0.169	22	3.02E-16	67.065
