# Supplementary material for: Causal Link between Inflammatory Bowel Disease and Fistula: Evidence from Mendelian Randomization Study
Source: J Clin Med. 2023 Mar 24;12(7):2482. doi: 10.3390/jcm12072482 (PMC10095427; doi:10.3390/jcm12072482)
Supplement: Supplementary file 1 [file jcm-12-02482-s001.zip › Supplementary table S3.pdf]

Supplementary table S3. (1) SNPs related to FISTULA; (2) 2 SNPs related to FISSANAL; (3) SNPs related to FEMGENFISTUL.

(1)

ID	SNP	Effect_allele	Other_allele	Se	Beta	Chr	P_val	F
finn-b-K11_FISTULA	rs10160633	G	A	0.0802	-0.378	11	2.41E-06	22.21
finn-b-K11_FISTULA	rs10889022	T	C	0.025	0.1529	1	9.19E-10	37.41
finn-b-K11_FISTULA	rs12961069	T	C	0.0255	0.1172	18	4.41E-06	21.12
finn-b-K11_FISTULA	rs13192019	C	T	0.2569	1.2612	6	9.12E-07	24.10
finn-b-K11_FISTULA	rs138578962	T	C	0.054	0.2647	19	9.51E-07	24.03
finn-b-K11_FISTULA	rs147730261	A	C	0.2168	1.0464	15	1.39E-06	23.30
finn-b-K11_FISTULA	rs17769930	T	C	0.0841	0.3882	12	3.93E-06	21.31
finn-b-K11_FISTULA	rs2634736	T	C	0.0368	-0.1913	3	2.01E-07	27.02
finn-b-K11_FISTULA	rs2701111	A	G	0.0215	0.1071	12	6.15E-07	24.81
finn-b-K11_FISTULA	rs2735178	C	T	0.0273	-0.1265	7	3.43E-06	21.47
finn-b-K11_FISTULA	rs34831382	G	A	0.0462	0.212	2	4.54E-06	21.06
finn-b-K11_FISTULA	rs35586534	G	A	0.0328	-0.1531	2	2.96E-06	21.79
finn-b-K11_FISTULA	rs577384250	A	G	0.0891	0.4334	10	1.16E-06	23.66
finn-b-K11_FISTULA	rs6124783	A	G	0.0258	0.1287	20	5.91E-07	24.88
finn-b-K11_FISTULA	rs61271555	A	G	0.0277	0.1322	11	1.85E-06	22.78
finn-b-K11_FISTULA	rs62506936	A	G	0.0535	-0.255	8	1.89E-06	22.72
finn-b-K11_FISTULA	rs6455970	G	A	0.0265	-0.1231	6	3.35E-06	21.58
finn-b-K11_FISTULA	rs72791105	T	G	0.0937	0.4338	2	3.71E-06	21.43
finn-b-K11_FISTULA	rs8074474	A	C	0.0496	0.2381	17	1.58E-06	23.04
finn-b-K11_FISTULA	rs9827246	T	C	0.022	0.1028	3	2.91E-06	21.83

(2)

ID	SNP	Effect_allele	Other_allele	Se	Beta	Chr	P_val	F
finn-b-K11_FISSANAL	rs10889022	T	C	0.0256	0.1592	1	5.20E-10	38.67
finn-b-K11_FISSANAL	rs11102109	C	A	0.0239	-0.1108	1	3.70E-06	21.49
finn-b-K11_FISSANAL	rs13187132	T	C	0.0318	-0.1466	5	3.93E-06	21.25
finn-b-K11_FISSANAL	rs13192019	C	T	0.2636	1.3107	6	6.61E-07	24.72
finn-b-K11_FISSANAL	rs138578962	T	C	0.0557	0.2764	19	6.87E-07	24.62
finn-b-K11_FISSANAL	rs17769930	T	C	0.0859	0.3947	12	4.33E-06	21.11
finn-b-K11_FISSANAL	rs2634736	T	C	0.0377	-0.1895	3	4.87E-07	25.27
finn-b-K11_FISSANAL	rs2701111	A	G	0.022	0.1118	12	3.77E-07	25.82
finn-b-K11_FISSANAL	rs55987116	A	C	0.0268	0.1385	20	2.33E-07	26.71
finn-b-K11_FISSANAL	rs577384250	A	G	0.092	0.4552	10	7.52E-07	24.48
finn-b-K11_FISSANAL	rs61271555	A	G	0.0284	0.1305	11	4.34E-06	21.11
finn-b-K11_FISSANAL	rs62506936	A	G	0.0549	-0.2696	8	9.17E-07	24.12
finn-b-K11_FISSANAL	rs6455970	G	A	0.0271	-0.1242	6	4.74E-06	21.00
finn-b-K11_FISSANAL	rs8074474	A	C	0.0509	0.2447	17	1.53E-06	23.11
finn-b-K11_FISSANAL	rs9827246	T	C	0.0225	0.1032	3	4.70E-06	21.04

(3)

ID	SNP	Effect_allele	Other_allele	Se	Beta	Chr	P_val	F
finn-b-N14_FEMGENFISTUL	rs10944369	T	C	0.0977	-0.4834	6	7.48E-07	24.48
finn-b-N14_FEMGENFISTUL	rs11005954	C	T	0.2293	1.0513	10	4.52E-06	21.02
finn-b-N14_FEMGENFISTUL	rs142466907	C	T	0.8182	4.1592	2	3.71E-07	25.84
finn-b-N14_FEMGENFISTUL	rs17160688	A	G	0.1693	0.8171	1	1.39E-06	23.29

finn-b-N14_FEMGENFISTUL	rs7765706	T	C	0.1001	-0.5447	6	5.33E-08	29.61
finn-b-N14_FEMGENFISTUL	rs7961226	C	T	0.1537	0.713	12	3.47E-06	21.52
