# Supplementary material for: Causal Link between Inflammatory Bowel Disease and Fistula: Evidence from Mendelian Randomization Study
Source: J Clin Med. 2023 Mar 24;12(7):2482. doi: 10.3390/jcm12072482 (PMC10095427; doi:10.3390/jcm12072482)
Supplement: Supplementary file 1 [file jcm-12-02482-s001.zip › Supplementary table S4.pdf]

Variable	Outcome						
	**Method**	**NSNP**	**OR (95% CI)**	**P_val**	**Heterogeneity p-value**	**Pleiotropy p-value**	**Mr_presso**
IBD to FISTULA							
	MR Egger	94	1.10 (1.00 to 1.21)	0.0481	0.1937	0.8636	0.2237
	Weighted median	94	1.10 (1.04 to 1.16)	0.0006			
	Inverse variance weighted	94	1.09 (1.05 to 1.13)	1.22E-06			
IBD to FISSANAL							
	MR Egger	94	1.12 (1.02 to 1.23)	0.0172	0.4448	0.6907	0.4804
	Weighted median	94	1.11 (1.05 to 1.17)	0.0001			
	Inverse variance weighted	94	1.10 (1.06 to 1.14)	6.12E-08			
IBD to FEMGENFISTUL							
	MR Egger	95	0.93 (0.75 to 1.16)	0.5359	0.8149	0.6484	0.8321
	Weighted median	95	0.95 (0.75 to 1.21)	0.6867			
	Inverse variance weighted	95	0.97 (0.85 to 1.11)	0.6688			
CD to FISTULA							
	MR Egger	76	1.03 (0.94 to 1.12)	0.5541	0.0093	0.3006	0.0113
	Weighted median	76	1.09 (1.04 to 1.13)	0.0001			
	Inverse variance weighted	76	1.07 (1.04 to 1.11)	3.13E-05			
CD to FISSANAL							
	MR Egger	76	1.02 (0.93 to 1.11)	0.6984	0.0142	0.1921	0.0116
	Weighted median	76	1.07 (1.02 to 1.12)	0.0042			
	Inverse variance weighted	76	1.08 (1.04 to 1.11)	1.89E-05			
CD to FEMGENFISTUL							
	MR Egger	76	1.01 (0.72 to 1.41)	0.9660	0.4885	0.8891	0.5138

	Weighted median	76	0.93 (0.76 to 1.14)	0.4856			
	Inverse variance weighted	76	0.99 (0.87 to 1.12)	0.8163			
UC to FISTULA							
	MR Egger	50	1.07 (0.93 to 1.23)	0.3350	0.2946	0.8919	0.3340
	Weighted median	50	1.07 (1.01 to 1.13)	0.0247			
	Inverse variance weighted	50	1.08 (1.04 to 1.12)	0.0001			
UC to FISSANAL							
	MR Egger	50	1.11 (0.97 to 1.27)	0.1278	0.4213	0.8316	0.4686
	Weighted median	50	1.10 (1.04 to 1.17)	0.0014			
	Inverse variance weighted	50	1.10 (1.06 to 1.14)	2.75E-06			
UC to FEMGENFISTUL							
	MR Egger	51	0.64 (0.39 to 1.04)	0.0794	0.2497	0.1309	0.2178
	Weighted median	51	0.80 (0.61 to 1.03)	0.0844			
	Inverse variance weighted	51	0.92 (0.77 to 1.09)	0.3159			
