# Supplementary material for: Causal Link between Inflammatory Bowel Disease and Fistula: Evidence from Mendelian Randomization Study
Source: J Clin Med. 2023 Mar 24;12(7):2482. doi: 10.3390/jcm12072482 (PMC10095427; doi:10.3390/jcm12072482)
Supplement: Supplementary file 1 [file jcm-12-02482-s001.zip › Supplementary table S5 .pdf]

Variable	Outcome						
	Method	NSNP	OR (95% CI)	P_val	Heterogeneity p-value	Pleiotropy p-value	Mr_presso
FISTULA to IBD							
	MR Egger	20	0.96 (0.89 to 1.03)	0.2302	0.5646	0.2794	0.5457
	Weighted median	20	0.97 (0.91 to 1.03)	0.3389			
	Inverse variance weighted	20	0.99 (0.94 to 1.03)	0.5482			
FISTULA to CD							
	MR Egger	19	0.98 (0.89 to 1.08)	0.6737	0.6604	0.5133	0.8021
	Weighted median	19	0.97 (0.89 to 1.05)	0.4059			
	Inverse variance weighted	19	0.99 (0.94 to 1.05)	0.8615			
FISTULA to UC							
	MR Egger	20	0.95 (0.87 to 1.04)	0.2695	0.6733	0.8506	0.7232
	Weighted median	20	0.95 (0.88 to 1.03)	0.2297			
	Inverse variance weighted	20	0.97 (0.92 to 1.03)	0.3260			
FISSANAL to IBD							
	MR Egger	15	0.96 (0.89 to 1.03)	0.2983	0.3900	0.7088	0.5064
	Weighted median	15	0.95 (0.88 to 1.02)	0.1513			
	Inverse variance weighted	15	0.97 (0.92 to 1.02)	0.2102			
FISSANAL to CD							
	MR Egger	15	0.98 (0.89 to 1.07)	0.6232	0.6733	0.8506	0.6559
	Weighted median	15	0.97 (0.88 to 1.06)	0.4925			
	Inverse variance weighted	15	0.98 (0.92 to 1.04)	0.5760			
FISSANAL to UC							

	MR Egger	15	0.94 (0.86 to 1.04)	0.2413	0.6604	0.5133	0.8004
	Weighted median	15	0.94 (0.86 to 1.03)	0.1911			
	Inverse variance weighted	15	0.95 (0.89 to 1.01)	0.0959			
FEMGENFISTUL to IBD							
	MR Egger	6	1.02 (0.99 to 1.06)	0.2478	0.1441	0.4252	0.2246
	Weighted median	6	1.01 (0.99 to 1.03)	0.2888			
	Inverse variance weighted	6	1.01 (0.99 to 1.03)	0.2702			
FEMGENFISTUL to CD							
	MR Egger	6	1.03 (1.00 to 1.07)	0.1295	0.1424	0.7641	0.6678
	Weighted median	6	1.02 (1.00 to 1.05)	0.1083			
	Inverse variance weighted	6	1.02 (1.00 to 1.04)	0.0348			
FEMGENFISTUL to UC							
	MR Egger	6	1.00 (0.96 to 1.05)	0.9083	0.1424		0.2937
	Weighted median	6	0.99 (0.96 to 1.02)	0.5284			
	Inverse variance weighted	6	1.00 (0.97 to 1.02)	0.7949			
