# Supplementary figures and images for: Causal Link between Inflammatory Bowel Disease and Fistula: Evidence from Mendelian Randomization Study
Source: J Clin Med. 2023 Mar 24;12(7):2482. doi: 10.3390/jcm12072482 (PMC10095427; doi:10.3390/jcm12072482)

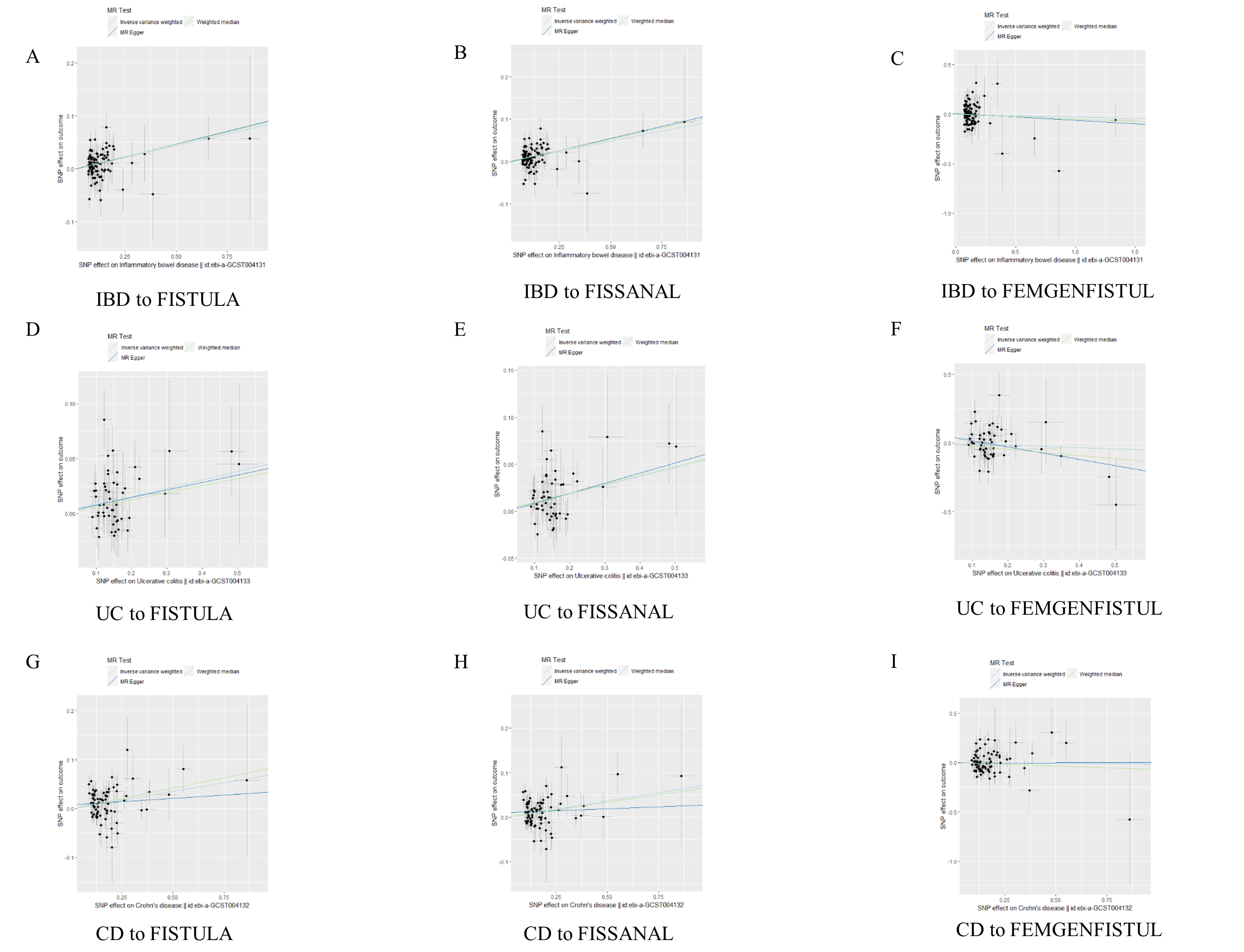

Supplement: Supplementary file 1 [file jcm-12-02482-s001.zip › Supplementary Figure S1.tif]

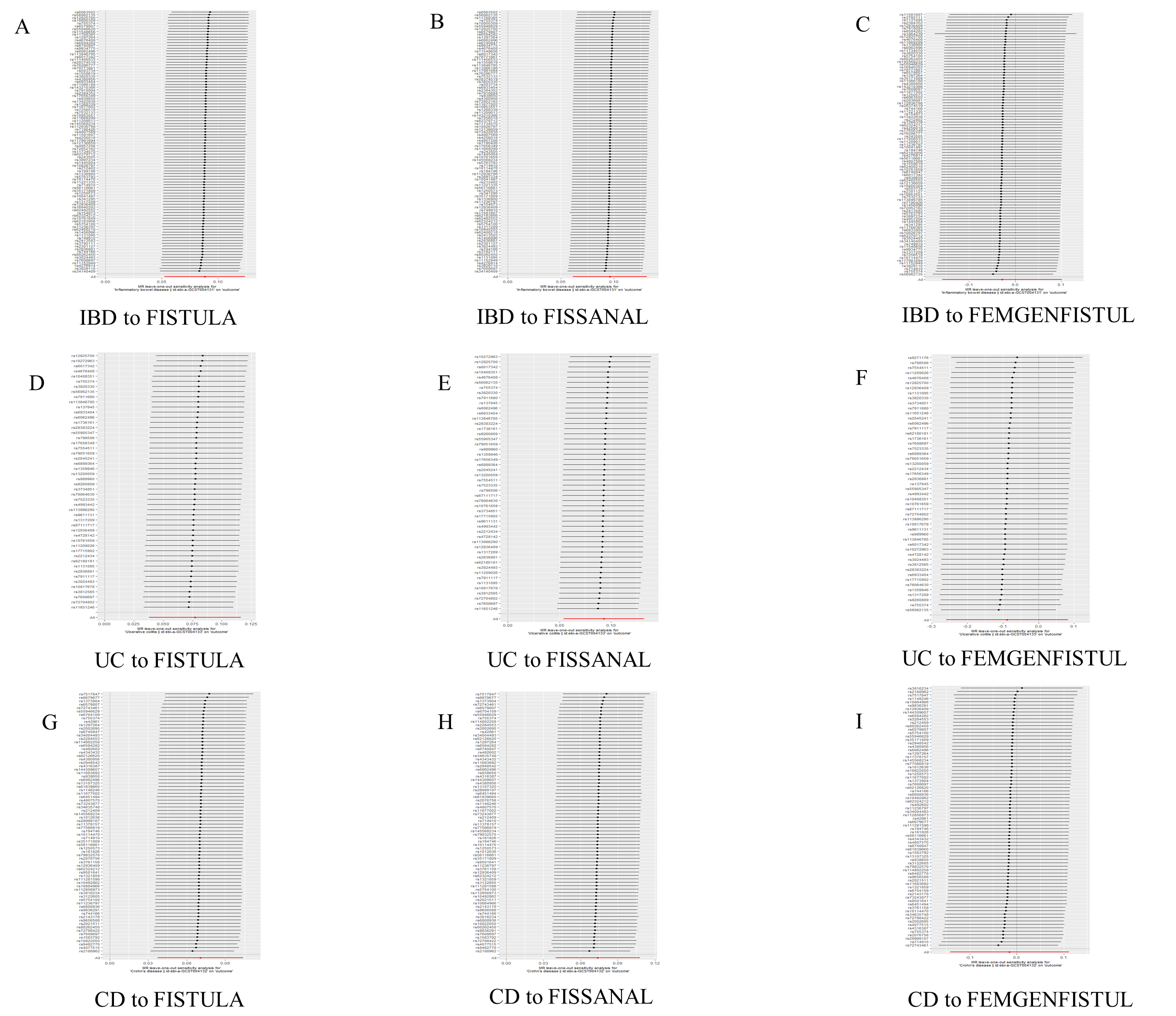

Supplement: Supplementary file 1 [file jcm-12-02482-s001.zip › Supplementary Figure S2.tif]

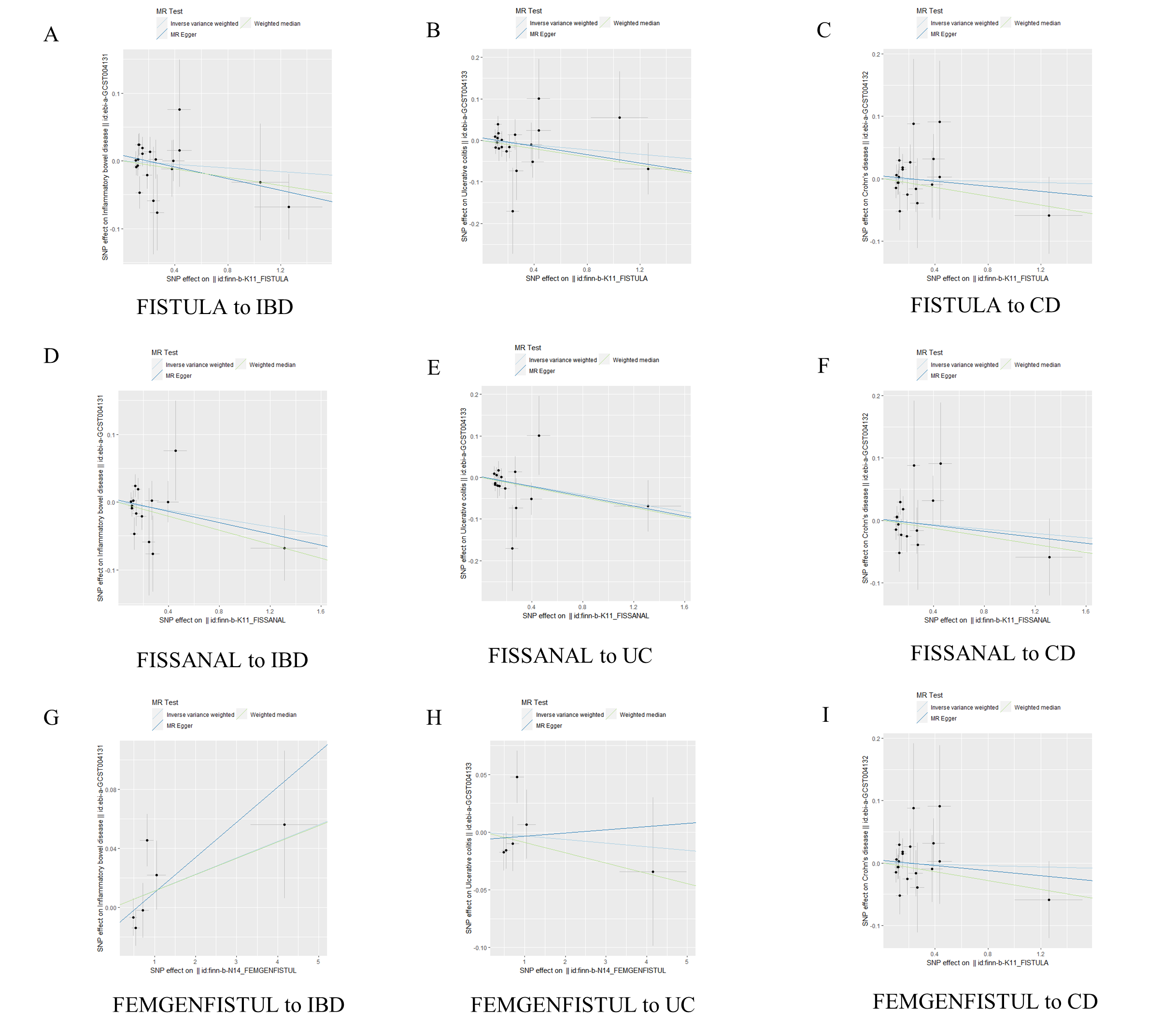

Supplement: Supplementary file 1 [file jcm-12-02482-s001.zip › Supplementary Figure S3.tif]

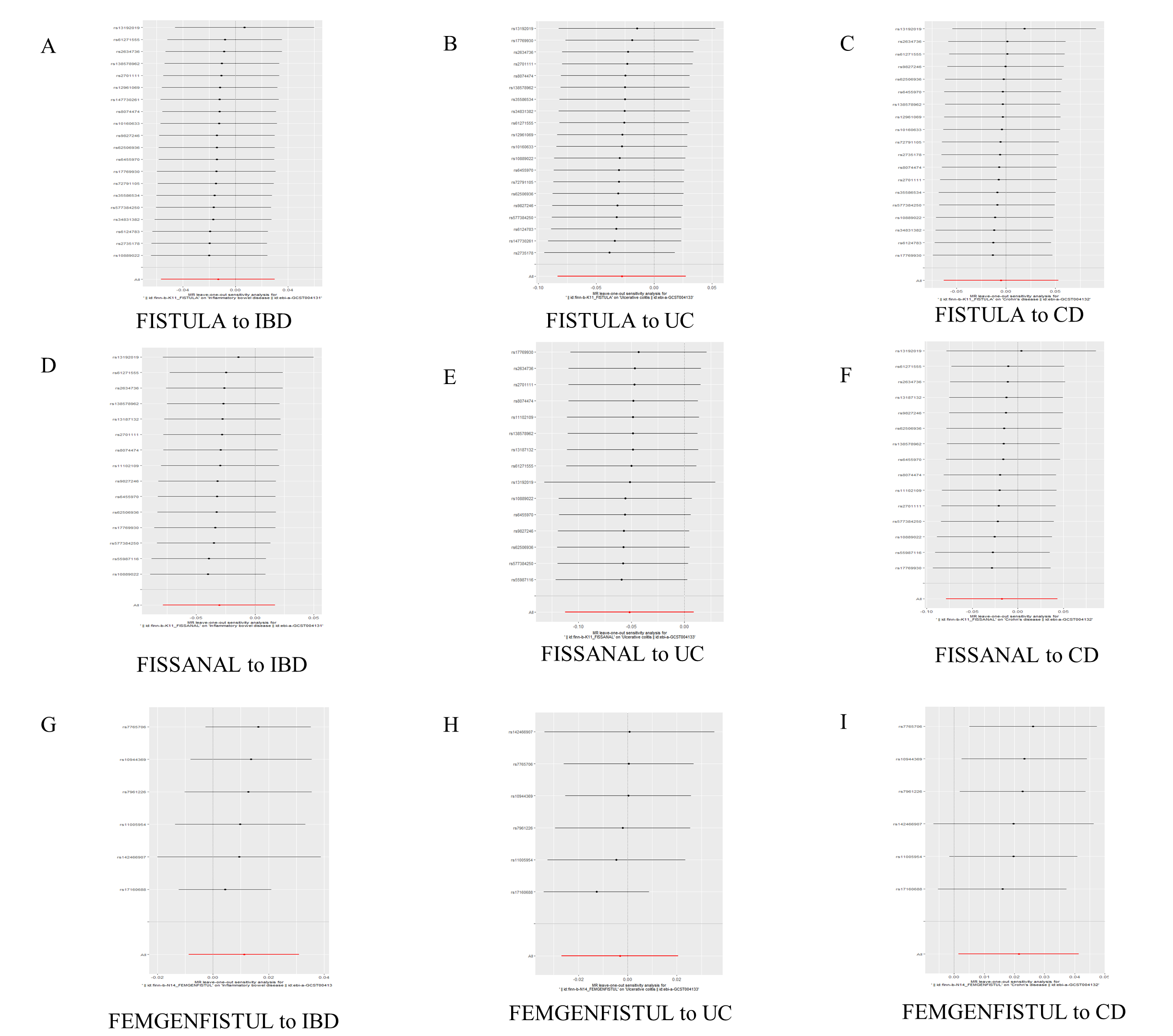

Supplement: Supplementary file 1 [file jcm-12-02482-s001.zip › Supplementary Figure S4.tif]
